# Augmented Renal Clearance: What Have We Known and What Will We Do?

**DOI:** 10.3389/fphar.2021.723731

**Published:** 2021-11-02

**Authors:** Yifan Luo, Yidan Wang, Yue Ma, Puxiu Wang, Jian Zhong, Yang Chu

**Affiliations:** ^1^ Department of Pharmacy, The First Hospital of China Medical University, Shenyang, China; ^2^ School of Pharmacy, China Medical University, Shenyang, China; ^3^ College of Food Science and Technology, Shanghai Ocean University, Shanghai, China

**Keywords:** augmented renal clearance, mechanism, clinical treatment, *in vivo* and *in vitro* model, therapeutic drug monitoring

## Abstract

Augmented renal clearance (ARC) is a phenomenon of increased renal function in patients with risk factors. Sub-therapeutic drug concentrations and antibacterial exposure in ARC patients are the main reasons for clinical treatment failure. Decades of increased research have focused on these phenomena, but there are still some existing disputes and unresolved issues. This article reviews information on some important aspects of what we have known and provides suggestion on what we will do regarding ARC. In this article, we review the current research progress and its limitations, including clinical identification, special patients, risk factors, metabolism, animal models and clinical treatments, and provide some promising directions for further research in this area.

## Introduction

**FIGURE 1:**
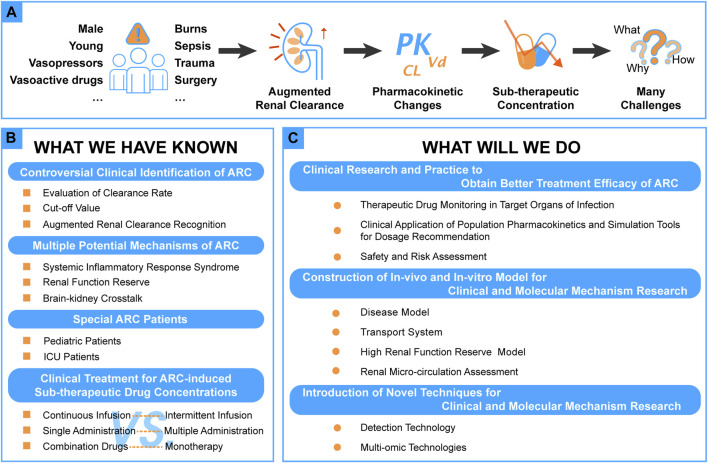
A schematic diagram of main content of this review.

Since Udy et al. proposed the concept of augmented renal clearance (ARC) in 2010 (Udy et al., 2010), the phenomenon of ARC and the individualization of pharmacotherapy has gradually attracted more attention recently (see Figure 1A).

ARC was defined as the enhanced renal elimination of circulating solutes compared to a baseline (Udy et al., 2010, 2011). In clinical practice, creatinine clearance (CL_cr_) ≥130 ml/min/1.73 m^2^ is usually considered a universally standard lower limit (Bilbao-Meseguer et al., 2018; Dhaese et al., 2021; Nicolau et al., 2021; Tang et al., 2021). ARC leads to sub-optimal drug exposure and causes treatment failure (Udy et al., 2010, 2011; Baptista et al., 2011; Baptista, 2018; Van Der Heggen et al., 2019; Avedissian et al., 2020). Although much attention has been given in this area, there still exist some questions, and some new experimental methods and techniques require exploration due to the complexity of the pathophysiological state of ARC.

There are many studies showed that the at-risk groups for ARC include younger patients, especially younger male patients, with lower illness severity scores on the Sequential Organ Failure Assessment (SOFA) or Acute Physiology and Chronic Health Evaluation (APACHE) II (Udy et al., 2013, 2017; Baptista et al., 2020; Nei et al., 2020; Saito et al., 2020; Johnston et al., 2021). Disease-related risk factors include trauma, surgery, sepsis, burn, subarachnoid hemorrhage, and hematological malignancy (Gerlach et al., 2019; Morbitzer et al., 2019; Lannou et al., 2020a; Saito et al., 2020). Patients who are faced with these disease-related factors usually owe their conditions to their underlying systemic inflammation states and receive resuscitation of large volumes of fluid, crystalloid and hypertonic saline solutions, and the administration of vasoactive drugs and vasopressors (Udy et al., 2013; Cook and Hatton-Kolpek, 2019; Beunders et al., 2020).

In this article, we review what we have known on some crucial aspects of ARC and discuss what we will do on ARC in the future. The aim is to provide a comprehensive understanding of ARC and supply some directions for further research in this area.

## What We Have Known

During the past decades, many studies related to ARC have been conducted and focused on clinical identification, possible mechanisms, ARC-induced sub-optimal concentrations, and corresponding ways of improvement (see Figure 1B).

### Controversial Clinical Identification of ARC

ARC focuses on two aspects of “augmented” and “renal clearance”, that is which methods are to be used to evaluate the kidneys’ function and how to judge the status of “augmented”. However, there are still controversies about the evaluation methods of renal function and the definition of ARC patients.

Many studies have shown that GFR measured by inulin excretion or radioactive tracer methods, the “gold standard”, are the most accurate methods for kidney function assessment (Soveri et al., 2014). There are other methods used for GFR assessment. Creatinine is regarded as the most common endogenous filtration marker and is detected over 24 h to evaluate the excretory function in “normal” conditions (Inker and Titan, 2021). But, a recent study (Collet et al., 2021) showed that compared with the measurement of GFR by iohexol clearance, the 6 h renal creatinine clearance systematically overestimated renal function in adult patients with ARC due to small muscle mass and nutrition, and the mean bias was higher than the calculated using formulas. Additionally, some convenient and simple methods have been developed for the rapid evaluation of kidney function, such as Cl_cr_ calculated by Cockroft-Gault (Cockcroft and Gault, 1976); eGFR calculated by the Chronic Kidney disease Epidemiology Collaboration (Levey et al., 2009) and Modification of Diet in Renal disease Study (MDRD) equations (Levey et al., 1999), which clinicians commonly adopt. But, there are still some issues, including that the calculation results are susceptible to many factors owing to unstable kidney function due to ARC and therefore underestimate renal function when identifying ARC (Baptista et al., 2014; Ruiz et al., 2015; Morbitzer et al., 2019; Gijsen M. et al., 2020). Therefore, more accurate methods and predictive equations for renal function estimation, high-risk screening, and the discovery of optimal surrogate markers are all needed for the rapid and straightforward recognition of ARC.

ARC is defined and recognized as CL_cr_ ≥ 130 ml/min/1.73 m^2^, based on numerous clinical studies finding that CL_cr_ ≥ 130 ml/min/1.73 m^2^ is related to target concentration attainment (Mahmoud and Shen, 2017), while some studies chose other cutoffs such as 120 ml/min/1.73m2, 150 ml/min/1.73m2 or else (Campassi et al., 2014; Carrié et al., 2019; Lannou et al., 2020b; Ong et al., 2021). Some researchers proposed that the ARC duration time should be carefully considered (Udy et al., 2014; Tomasa-Irriguible et al., 2021). In a study of GFR estimation on critically ill patients, conducted by Baptista’s team (Baptista et al., 2014), “ARC patient” were defined as ≥50% measurements of CL_cr_ ≥ 130 ml/min/1.73 m^2^ during the admission period. And Claus et al. (Claus et al., 2013) found that patients who permanently expressed ARC during antimicrobial treatment had higher treatment failure rates (33.3 vs 17.4%) than patients with transient ARC (1 day). Some exploration has also been done for screening and recognizing ARC patients by using scoring systems (Morbitzer et al., 2019; Gijsen M. P. et al., 2020; Saito et al., 2020). But the risk factors screening and the cutoff value were variant in different studies. Udy’s team (Udy et al., 2013) described a model of age, modified SOFA, and diagnostic category, which was used to predict patients manifesting ARC. In 2016, Barletta and others (Barletta et al., 2017) described a predictive model of ARC, which is specific to the intensive care unit (ICU) trauma patients for bedside application and ARCTIC scores ≥6 presented as the cutoff for ARC. Thus, a unified standard of ARC containing cutoff, duration time or scoring criteria should be clearly defined.

### Multiple Potential Mechanisms of ARC

**FIGURE 2:**
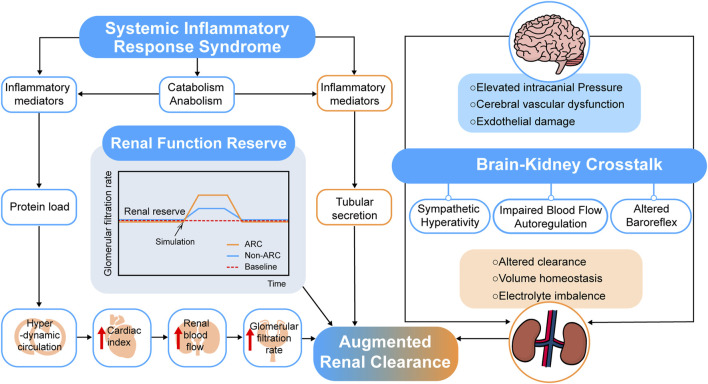
Overview of probable mechanisms of augmented renal clearance.

The mechanism of ARC is not clear up to now (Bilbao-Meseguer et al., 2018; Baptista et al., 2019) due to the hyperkinetic state, increased cardiac output, and elevated blood flow to major organs of patients at risk of manifesting ARC. Systemic inflammatory response syndrome (SIRS) and renal function reserve (RFR) were proposed to explain the possible mechanisms of ARC (see Figure 2).

The theory of SIRS posed that when patients in conditions such as severe trauma, burns, sepsis and major surgery related or irrelevant to infection, cytokines and pro-inflammatory mediators release, which may decrease vascular resistance and increase cardiac output and capillary permeability (Balk, 2014). For critically ill patients, the use of many fluids and positive inotropic drugs for treatment also makes the renal vascular flow further increase, leading to the occurrence of ARC (Sime et al., 2015). A prospective observational study of COVID-19 (Beunders et al., 2021) showed that the detected time point of ARC was strongly related to the day of peak ferritin, C-reactive protein, and D-dimer. The research team of Udy (Udy et al., 2013) has conducted a prospective observational study in 71 patients with sepsis and multi-trauma, 57.7% of whom manifested with ARC, and the results showed that there was a weak correlation between cardiac index (CI) and CL_cr_ (r = 0.346; *p* = 0.003). Further, changes in vascular resistance and capillary permeability as well as the influence of inotropic drugs are still unclear and need to be verified by more experiments.

Another theory of RFR suggests that renal reserve plays a role in ARC. RFR refers to the capacity of kidneys under certain physiological conditions or pathological stimuli, such as pregnancies, high-protein diets, high fluid intakes, or uses of high cardiac output drugs (Sharma et al., 2014). The stress tests showed that vasodilation and increased blood flow might be the mechanisms due to the release of endothelium-derived relaxation and prostaglandins locally, displayed as an increase in GFR. Additionally, younger patients tend to have higher renal reserve and function (Ronco et al., 2017). This theory is consistent with the risk factors of ARC, which increases the possibility of the veracity of the RFR theory.

In addition to these two theories, other studies conjectured possible mechanisms of ARC. Dias et al. (Dias et al., 2015) tried linking renal function with traumatic brain injury, and the results showed that PRx (intracranial pressure, cerebral perfusion pressure, and the cerebrovascular reactivity pressure index) was significantly related to ARC in traumatic brain injury patients. A “brain-kidney crosstalk” theory was proposed by Nongnuch et al. (Nongnuch et al., 2014) in an AKI study, and Khalid et al. (Khalid et al., 2019) also posed that traumatic brain injury-resulted crosstalk between the brain and kidney cause chaotic perturbations and affect their perfusion regulation, enlightened us to a possible link between acute brain injury and kidney function, which still requires further proof in ARC patients (see Figure 2).

### Special ARC Patients

#### Pediatric Patients

The modified Schwartz equation is most adopt for evaluating renal function in pediatric patients (Bauters et al., 2019; Gao et al., 2020). There are ten different ARC definitions (CL_cr_ ≥ 130–250 ml/min/1.73 m^2^) in pediatrics, although the cutoff commonly defined as the same as adults (CL_cr_ ≥ 130 ml/min/1.73 m^2^) (Rhoney et al., 2021). The reported risk factors for pediatric ARC patients include serum creatinine, age, febrile neutropenia, male, septic shock, and antibiotic treatment (Ishii et al., 2018; Van Der Heggen et al., 2019). But increased complexity and difficulty for ARC assessment is in pediatric patients than adults due to rapid growth of the body with age and maturity of kidney and other organs (Rodieux et al., 2015).

#### ICU Patients

Patients in the ICU always undergo multiple organ failure, illness severity, hemodynamic instability, and exposure to the amount of fluid resuscitation, and more susceptible to nosocomial infections which may lead to comorbidities and complications caused by pneumonia related to mechanical ventilation and infections following trauma or surgery (Eggimann and Pittet, 2001; Vanhorebeek et al., 2020). The evaluation of ARC in ICU patients is more complicated because of these multiple influencing factors.

### Clinical Treatment for ARC-Induced Sub-therapeutic Drug Concentrations

Compared to patients with normal kidney function, ARC patients have higher clearances and shorter half-lives of drugs, which promote drug concentrations to fall rapidly. Standard doses make it challenging to meet treatment requirements in these cases (Kaska et al., 2018; Chen and Nicolau, 2020). Some studies have attempted to obtain sufficient drug exposure by changing the way of infusion, altering the frequency of administration, or using drug combinations.

#### Continuous Infusion vs. Intermittent Infusion

For many drugs such as β-lactams and antiepileptic drugs (e.g., levetiracetam), therapeutic drug monitoring (TDM) is recommended to reach adequate drug exposure. Compared to the intermittent infusion (II), continuous infusion (CI) prolonged T > MIC to achieve pharmacodynamic targets and obtain a higher clinical cure rate (Carrié et al., 2018; Kondo et al., 2020; Goncette et al., 2021). Further, pharmacokinetic/pharmacodynamic (PK/PD) studies have shown that continuous infusion can also increase the probability of target attainment (Roberts et al., 2016; Barrasa et al., 2020).

#### Single Administration vs. Multiple Administration

Based on the importance of achieving a high bactericidal concentration of aminoglycoside in the initial stage of anti-infective treatment in an ICU (Aréchiga-Alvarado et al., 2020), a once daily dose of amikacin showed a better effect than multiple doses, daily.

#### Combination Drugs vs. Monotherapy

It was found that under ARC conditions, the intermittent dosing regimen of meropenem and ciprofloxacin as monotherapy is not effective against *Pseudomonas aeruginosa*, even at the maximum approved daily dose for sensitive strains. However, the combination of the intermittent dosing regimen could effectively suppress organisms (Agyeman et al., 2021).

## What We Will Do

Although many studies have been conducted in the field concerning ARC, there are still some unknown areas in need of further research. In this section, some advanced research methods are provided for further research in this area (see Figure 1C).

### Clinical Research and Practice to Obtain Better Treatment Efficacy of ARC

The “hyper-dynamic” circulation state of ARC leads to increased renal delivery and elimination of drugs causes sub-therapeutic drug concentrations and sub-optimal antibacterial exposures in ARC patients who are the main reasons for clinical treatment failure (Cook and Hatton-Kolpek, 2019; Tomasa-Irriguible et al., 2020). So there are still some issues should be explored by research or clinical practice, which will provide valuable references to clinical drug therapy to clinicians and clinical pharmacists in the future.

#### Therapeutic Drug Monitoring in Target Organs of Infection

As a routine clinical test method, TDM can be used to monitor drug concentration in ARC patients. Researches showed that the pharmacokinetic behavior of ARC patients has been changed. The typical values of clearance (CL) and volume of distribution (V) of vancomycin in ARC patients were 8.515 L/h and 2.22 L/kg, which were higher than in the population of normal renal function reported previously (Chu et al., 2020). Some other studies have also found that the CL and V of ceftolozane and tazobactam in ARC patients were both higher than in the healthy subjects (Nicolau et al., 2021). These results pointed out that drugs were distributed widely in patients manifesting ARC, which reminded us that it benefits the treatment of infections in tissues with weak drug penetration or cause unexpected toxicity. But in most centers, serum or plasma drug concentrations are sampled and detected as a surrogate due to practical limitations, which do not reflect the real concentrations at the sites of infection.

So, it is preferred to conduct TDM in target organs of infection, such as cerebrospinal fluid in meningitis and epithelial lining fluid in pneumonia, which will predict and explain the clinical treatment response better (Felton et al., 2018; Abdul-Aziz et al., 2020; McCreary et al., 2020).

#### Clinical Application of Population Pharmacokinetics and Simulation Tools for Dosage Recommendation

In view of ARC patients, the dosage regimens need to be optimized in clinical therapy. Tools such as population pharmacokinetics (PPK) and Bayesian estimators combined with other simulation means (e.g., JPKD, Smartdose, Vancomycin Calculator, Monte Carlo simulations) have been used to predict individual pharmacokinetic parameters and to yield clinical dosage recommendations including many antibiotics that are commonly used in clinical practice, such as acyclovir and valacyclovir, linezolid, vancomycin, cefathiamidine, and levetiracetam in patients with ARC (Abdalla et al., 2020; Barrasa et al., 2020; Chu et al., 2020; Gijsen M. P. et al., 2020; Lv et al., 2020; Du et al., 2021; Ong et al., 2021).

The issue is that dosage regimens of ARC patients proposed by pharmacokinetic simulation software are always higher than empirical doses to achieve PK/PD targets (Mahmoud and Shen, 2017; Abdalla et al., 2020; Wang et al., 2021), which might be viewed with caution and have hardly been promoted in clinical practice.

In the follow-up research, more large-scale multi-center PPK studies should be performed to understand the influence factors of ARC in-depth and provide more accurate data for adjusting ARC treatment regimen.

#### Safety and Risk Assessment

Higher doses for ARC patients are always required to obtain sufficient drug exposure, so the safety and risk assessment should not be ignored.

There are some studies have reported the adverse reactions in ARC patients. In the study of vancomycin administration in patients with different renal function statuses (Yu et al., 2021), the adjusted daily dose of vancomycin in ARC patients was 2.8g/day, higher than the normal renal function group. After treatment with vancomycin, there were 14 cases of ARC changed into normal renal function (NRF) and 3 cases of ARC that changed into impaired renal function (IRF). It has also been reported that acute kidney injury has happened in three ARC patients during vancomycin therapy (1.0–1.5 g, bid), mainly caused by the combined use of nephrotoxic drugs (mannitol and etimicin) and ischemic injury of insufficient renal perfusion (LU et al., 2019).

The main question is that we still lack experience in dosage regimen formulation and adjustments for ARC patients, leading to the safety and risks are still unclear. So in the next step, the evaluation should be performed through multi-center prospective researches, in which factors including combinations of medications, alternatives of tissue toxicity, drug permeability should all be taken into consideration, providing desirable effectiveness and confirmable safety dosing regimens for patients manifesting ARC.

### Construction of *In-vivo* and *In-vitro* Models for Clinical and Molecular Mechanism Research

Since the mechanism of ARC is not yet clear, there are only limited animal studies on ARC have been reported. In these studies, iohexol and *p*-aminohippuric acid (PAH) were detected in blood as potential markers to evaluate the GFR, effective renal plasma flow and tubular secretion (Dhondt et al., 2020; Stroobant et al., 2020). Dhondt et al. used lipopolysaccharides (LPS) by continuous infusion to induce a sepsis piglets model, and elevated clearances of GFR marker iohexol and exogenously creatinine and effective renal plasma flow (ERPF) marker PAH were observed (Dhondt et al., 2021b). Decreased systemic exposures of iohexol and amikacin were found after fluid administration, suggesting that fluid therapy is a key factor involved in the development of ARC (Dhondt et al., 2021a).

Considering the mechanism of ARC is unclear till now, animal models based on the risk factors of ARC, including sepsissubarachnoid hemorrhage, burns, and high RFR or modeled by injection of amino acids to stimulate the disease state and augmented GFR (Ge et al., 2019; Lagier et al., 2019; Hu et al., 2020) may be better research method to explore ARC mechanism. Moreover, in the following research, *in vivo* and *in vitro* models such as isolated kidney perfusion, transporter knockout mice for transport system studies on drugs with different excretion mechanisms (Higgins et al., 2012; Ma et al., 2018) and invasive or noninvasive techniques such as synchrotron radiation, fluorescence microangiography methods, and intravital multiphoton microscopy for renal micro-circulation assessment (Li et al., 2021), will help us explore the mechanism and deeply understand the occurrence and development process of ARC.

### Introduction of Novel Techniques for Clinical and Molecular Mechanism Research

There are still some uncertainties and unknown areas in the field of ARC research due to a lack of effective research technologies and methods. With the emergence of new detection technology and advanced analytical methods applied in clinical research, the occurrence and development of diseases and their mechanisms can be intensively investigated and deeply understood. Application of ultra-performance liquid chromatography coupled with quadrupole time-of-flight mass spectrometry (UPLC-QTOF-MS), immuno-histochemical staining, multi-omic technologies (metabolomics, proteomics, genomics, lipidomics) research based on biological samples, differential gene expression analysis, biological pathway enrichment analysis, biological function analysis, and other methods will help us obtain and reveal the critical biomarkers, key pathways, and possible pathogenesis of ARC (Guan et al., 2020; Pang et al., 2020; Fukumura et al., 2021).

## Summary and Outlook

The causes of ARC concern a series of endogenous and exogenous factors which lead to elevated levels of GFRs and the hyperperfusion of drugs. Simple and accurate methods and standard cutoff values are still needed to define ARC, and the duration of transient or permanent expression of ARC is still an unresolved argument. Additionally, the mechanisms of ARC are complex and are not presently clear. Further, doses and medical regimens for treatment choices in ARC patients are facing big challenges. The mechanism of ARC lays the groundwork for the subsequent studies, so optional new technologies such as integrative omics analysis can be performed to explore the differences of metabolites and regulatory genes between ARC and non-ARC patients, which can clarify the mechanism of ARC. The *in vitro* and *in vivo* model of ARC can also be established and employed for a deep investigation into ARC base on the findings of the mechanism. Clinically applicable and practical therapeutic schedules of ARC patients ought to be explored and verified through large-scale multi-center researches.
